# Why and when should organisms elongate their telomeres? Elaborations on the ‘excess resources elongation’ and ‘last resort elongation’ hypotheses

**DOI:** 10.1002/ece3.10825

**Published:** 2023-12-13

**Authors:** David Gómez‐Blanco, Michael Tobler, Dennis Hasselquist

**Affiliations:** ^1^ Department of Biology Lund University Lund Sweden

**Keywords:** critical threshold in telomere length, individual strategies, life‐history trade‐offs, telomere elongation, telomere maintenance, telomere restoration

## Abstract

Telomere length and telomere shortening are thought to be critical cellular attributes and processes that are related to an individual's life span and fitness. The general pattern across most taxa is that after birth telomere length gradually decreases with age. Telomere protection and restoration mechanisms are usually assumed to reduce the rate of shortening or at most keep telomere length constant. However, here we have compiled a list of 26 articles showing that there is an increasing number of studies reporting apparent elongation of telomeres (i.e., a net increase in TL from time_*t*_ to time_*t*+1_) often in a considerable proportion of the individuals studied. Moreover, the few studies which have studied telomere elongation in detail show that increases in telomere length are unlikely to be due to measurement error alone. In this article, we argue that episodes of telomere elongation deserve more attention as they could reflect individual strategies to optimise life histories and maximise fitness, which may not be reflected in the overall telomere dynamics patterns. We propose that patterns of telomere (net) elongation may be partly determined by other factors than those causing telomere shortening, and therefore deserve analyses specifically targeted to investigate the occurrence of telomere elongation. We elaborate on two ecological hypotheses that have been proposed to explain patterns of telomere elongation (the ‘excess resources elongation’ and the ‘last resort elongation’ hypothesis) and we discuss the current evidence for (or against) these hypotheses and propose ways to test them.

## Graphical abstract

Telomere length can contribute to an individual's life span and fitness. Telomere length gradually decreases with age, however, elongation of telomeres can occur. Telomere elongation could reflect individual strategies to optimise life histories and maximise fitness. We elaborate on the ‘excess resources elongation’ and the ‘last resort elongation’ hypothesis to explain patterns of telomere elongation, reviewing their current support and proposing ways to test them.

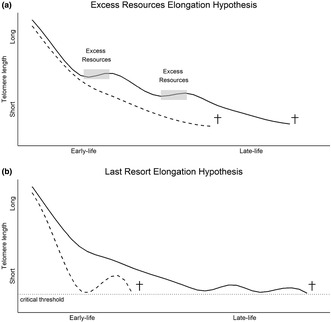

## 1 INTRODUCTION

Senescence is generally described as the time‐dependent accumulation of somatic damage and is eventually expressed as general somatic vulnerability, impaired somatic functions and degenerative diseases that ultimately may cause death (Gaillard & Lemaître, 2020; Van Deursen, 2014). To avoid or repair such damage, thus preserving the integrity of the body, organisms invest energy and nutritional resources in several somatic maintenance processes. One fundamental process for the viability of cells that undergo proliferation and differentiation is telomere maintenance and restoration (Blackburn et al., 2006; Shay & Wright, 2019). Telomeres consist of multiple repeats of a non‐coding DNA sequence (TTAGGG in vertebrates) that is located at the ends of linear chromosomes to prevent chromosome malfunctions, such as degradation and chromosome fusions (Blackburn, 2005; Monaghan, 2010). During each cell division, a fraction of the telomeric repeats at the ends of the chromosomes cannot be replicated, viz. the ‘end replication problem’, and telomeres therefore gradually shorten over an organism's life (Wellinger, 2014). In addition, cells also suffer telomere shortening when exposed to different stressors, including oxidative stress (Haussmann & Marchetto, 2010; Muraki et al., 2012; Reichert & Stier, 2017; but see, Boonekamp et al., 2017), psychological stress (Epel et al., 2004; Kiecolt‐Glaser & Glaser, 2010) and infections (Asghar et al., 2015; Effros, 2011; Ilmonen et al., 2008). Eventually, when a critical (short) telomere length is reached (i.e., when reaching a ‘critical threshold’; Monaghan, 2010; Hasselquist & Tobler, 2021; Tobler et al., 2022) further shortening will lead to cell senescence and apoptosis, which may contribute to organismal ageing (Cawthon et al., 2003; López‐Otín et al., 2013; Stewart & Weinberg, 2000). Short TL has been linked to an increased risk of several disorders and diseases (e.g., bone marrow failure, pulmonary fibrosis) and some cancers (Stanley & Armanios, 2015; Ujvari et al., 2022) with ultimate negative effects on health and longevity. Therefore, it may be beneficial for individuals to invest in telomere restoration (i.e., the addition of telomeric repeats at the ends of chromosomes) to prevent the cellular problems that appear when telomeres shorten to a critical length. In this article, we draw attention to a specific type of restoration, namely the transient lengthening of telomeres (i.e., telomere elongation) that appears to occur in a considerable fraction of individuals in rather many of the studied taxa (see our compilation of 26 studies showing telomere elongation in Table 1). We propose that episodes of telomere elongation could be adaptive and reflect individual strategies to optimise life history decision and maximise fitness. To extend the current theoretical framework for the occurrence of adaptive telomere elongation, we here summarise some data from studies that have found apparent telomere elongation and elaborate on the two hypotheses that have proposed adaptive explanations for the occurrence of telomere elongation.

**TABLE 1:** Illustration of the frequency of telomere elongation in experimental observational studies.

Study	*N*	% of elongation	Sampling interval	Tissue	Method	Taxa	Species	Life stage	Experimental manipulation?
Canestrelli et al. (2021)	36	75	1 year	RBC	qPCR	Amphibian	Tyrrhenian tree frog	Adult	No
Pauliny et al. (2012)	34	47	2 years	Hole blood	TRF	Bird	Barnacle goose	Adult	No
Parolini et al. (2015)	60	39	9 days	RBC	qPCR	Bird	Barn swallow	Nestling	No
Salmón et al. (2017)	49	61	1 year	RBC	qPCR	Bird	Great tit	Juvenile to Adult	No
Spurgin et al. (2018)	655	44	1–15 years	Hole blood	qPCR	Bird	Seychelles warbler	Juvenile to Adult	No
Stier et al. (2020)	107	5	20–40 days	RBC	TRF	Bird	Japanese quail	Embryo	Yes
Brown et al. (2021)	359	46	1–15 years	Hole blood	qPCR	Bird	Seychelles warbler	Juvenile to Adult	No
Wolf et al. (2021)	147	48	7 days	Hole blood	qPCR	Bird	Tree swallows	Nestling	Yes
Sheldon et al. (2022)	150	45	1–10 years	Hole blood	qPCR	Bird	Zebra finch	Adult	No
Sheldon et al. (2022)	50	34	8 days	Hole blood	qPCR	Bird	Zebra finch	Nestling	No
Gómez‐Blanco (2023)	209	13	1 year	Hole blood	qPCR	Bird	Great reed warbler	Juvenile to Adult	No
Le Pepke et al. (2023)	310	41	269 days	Hole blood	qPCR	Bird	House sparrows	Juvenile to Adult	No
Voirin et al. (2023)	364	65	6 days	RBC	qPCR	Bird	Barn swallow	Nestling	No
Xiong (2023)	98	62	30 days	RBC	qPCR	Bird	Zebra finch	adult	Yes
McLennan et al. (2017)	18	11	1 year	Fin tissue	qPCR	Fish	Atlantic salmon	Juvenile to Adult	No
Gardner et al. (2005)	70	9	1–13 years	Leukocytes	TRF	Mammal	Human	Young adult	No
Aviv et al. (2009)	561	12	10 years	Leukocytes	TRF	Mammal	Human	Adult	No
Nordfjäll et al. (2009)	959	34	10 years	Leukocytes	qPCR	Mammal	Human	Adult	No
Shalev et al. (2013)	236	17	5 years	Leukocytes	qPCR	Mammal	Human	Child	No
Steenstrup, Hjelmborg, Mortensen, et al. (2013)	80	11	10 years	Leukocytes	TRF	Mammal	Human	Adult	No
Huzen et al. (2014)	5886	34	1 year	Leukocytes	qPCR	Mammal	Human	Adult	No
Hoelzl et al. (2016)	50	50^a^	90 days	Leukocytes	qPCR	Mammal	Edible dormice	Adult	Yes
van Lieshout et al. (2019)	449	61	1–13 years	Leukocytes	qPCR	Mammal	European badger	Juvenile to Adult	No
Huang et al. (2021)	4053	22	8 years	Leukocytes	qPCR	Mammal	Human	Old adult	No
Ujvari et al. (2017)	40	35	2 years	Leukocytes	qPCR	Reptile	Frillneck lizard	Juvenile to Adult	No
Fitzpatrick et al. (2021)	40	70	1 year	Tail tissue	qPCR	Reptile	Spotted snow skink	Juvenile to Adult	No

*Note*: We carried out a non‐exhaustive literature search of studies conducted with different methods and in different vertebrate species and life stages. Year = publication year; *N* = sample size; % of elongation = percentage of individuals with elongated TL in the case study; Sampling interval = time gap between samplings; Tissue = biological material used in the study to measure TL (RBC; red blood cells); Method = TL measurement procedure; Life stage = developmental stage of the individuals in the study.

^a^ Values estimated from figures.

## 2 MINIMISING TELOMERE SHORTENING VERSUS INVESTING IN TELOMERE ELONGATION

**FIGURE 1:**
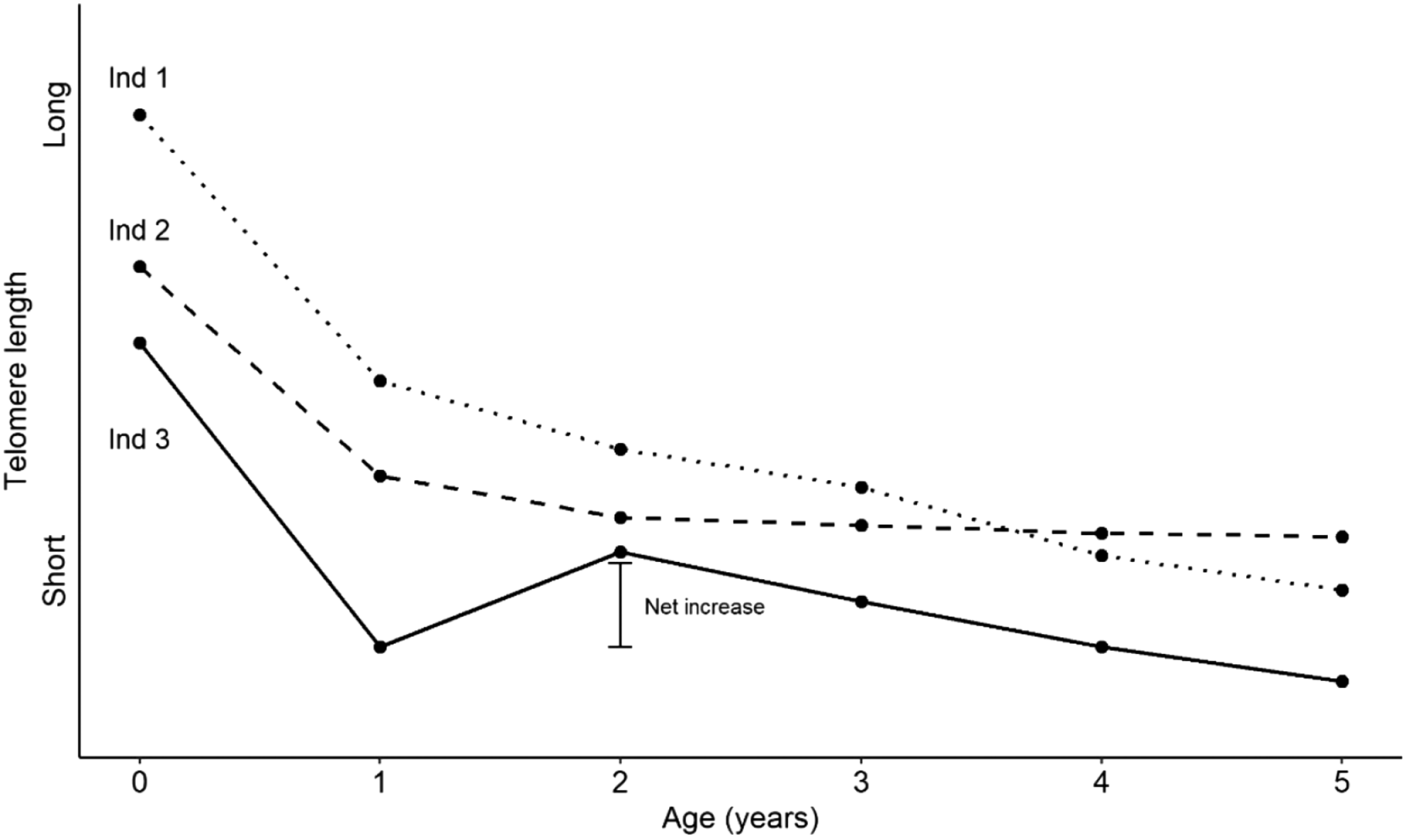
Fictive example of relationship between age (years) and telomere length (TL) in three individuals. The general long‐term pattern is that telomeres shorten over life, although we can distinguish different telomere dynamics patterns. Individual 1 undergoes continuous TL shortening over life. In individual 2, on the other hand, TL remains constant over time after 1 year of age (suggesting that TL is maintained at similar level due to the actions of telomere protection and telomere restoration mechanisms). In contrast, individual 3 invests even more in telomere restoration processes from 1 to 2 years of age, and this results in a temporary TL elongation (i.e., it reverses the shortening process, thus causing a net increase in TL between 1 and 2 years of age).

It is well documented for many taxa that telomeres gradually shorten over the life span (i.e., telomere shortening; exemplified in Figure 1, Ind 1). Moreover, the long‐held view is that telomere protection and restoration mechanisms (i.e., telomere maintenance) only reduce the rate of shortening or, at most, keep TL fairly constant (Lansdorp, 2008; Monaghan, 2010; Shay & Wright, 2007; Taylor & Delany, 2000), as illustrated by Ind 2 in Figure 1. However, there is an increasing number of studies on a wide range of taxa suggesting that lengthening of telomeres (i.e., an increase in telomere length between two time points) may occur during different periods of life in a non‐negligible proportion of individuals of a population (Table 1). Such cases of telomere elongation have often been interpreted as measurement errors (e.g., Chen et al., 2011; Steenstrup, Hjelmborg, Kark, et al., 2013). However, recent studies show that measurement error alone is unlikely to explain patterns of telomere elongation (Bateson & Nettle, 2017; van Lieshout et al., 2019; Voirin et al., 2023; Wolf et al., 2021). Given that elongation appears to be a real biological phenomenon, we therefore argue that (periodic) lengthening may be an important aspect of overall telomere dynamics (see also Epel, 2012). Telomere elongation is different from the ordinary telomere maintenance processes as it (temporarily) reverses the telomere shortening process and results in a net increase in TL from time_*t*_ to time_*t*+1_ (Figure 1, Ind 3). It may be seen as an extra investment in somatic maintenance that could be advantageous because longer telomeres have been shown to be associated with improved somatic condition, increased survival prospects and, hence, might result in higher Darwinian fitness (Heidinger et al., 2012; Monaghan, 2010; Olsson et al., 2018; Young et al., 2021). Telomere elongation may help cells with short telomeres regain their proper function, including tissue regeneration and repair, which contributes to the overall health and well‐being of the organism (Blackburn et al., 2006; Shay & Wright, 2019). Finally, elongation of telomeres has the potential to promote longevity and reduce the risk of age‐related diseases like cancer, heart problems and neurodegenerative conditions (López‐Otín et al., 2013; Samani et al., 2001; Sampson & Hughes, 2006). However, elongating telomeres may also entail severe costs. It has for example been argued that activating telomerase (a polymerase that builds and attaches new telomeric repeats to the chromosomal ends, see below) could entail several types of non‐negligible costs (Casagrande & Hau, 2019; Herborn et al., 2014; Nettle et al., 2015; Young, 2018). One type of short‐term physiological cost may be a ‘resource cost’ (reviewed in Young, 2018). The need to allocate limited resources (e.g., energy or nutrients) into the protection and restoration, including elongation, of telomeres may have to be traded off against other resource‐demanding functions (e.g., heat production or reproduction), leading to an adaptive ‘acceptance’ of telomere shortening (Casagrande & Hau, 2019; Young, 2018). Another type of cost may be the potentially large (long‐term) cost of activating the TL restoration machinery which is thought to result in an increased risk of cell immortalization facilitating the development of malign cancers (De Lange & Jacks, 1999; Holt et al., 1996; Shay, 2016; Young, 2018). It has been hypothesised that there is a trade‐off between the benefits of telomere restoration and the cost of increasing the risk of cancer. The outcome of this trade‐off may be that the direct benefit of cancer suppression during the reproductively active stages of life outweighs the long‐term cost of age‐related pathologies associated with short TL later in life, in agreement with antagonistic pleiotropy models (Caulin & Maley, 2011; Gomes et al., 2011; Kirkwood & Holliday, 1979).

The existence of telomere restoration‐associated life‐history trade‐offs, e.g., between delayed senescence benefits and resource/cancer costs, is expected to result in among‐individual variation in life‐history strategies (Monaghan, 2010; Tobler et al., 2022; Young, 2018). We suggest that studies specifically devoted to investigating patterns of telomere elongation can provide further insights into the mechanistic underpinnings of life‐history trade‐offs and how selection might act on such trade‐offs. We predict that individuals should invest differently in telomere restoration depending on the selection pressures they are facing (e.g., harsh environmental conditions, reproductive costs, infections) as well as on their current physiological state (e.g., physiological condition or telomere length). Here, we argue that even though in the perspective of an organism's whole life the general pattern is telomere shortening, episodes of telomere elongation during certain periods of life may be seen as larger investments that may either; (i) compensate for some of the previous losses in TL, (ii) prepare the organism for stressful periods to come in the future or (iii) be used as a ‘last resort’ to move a short TL further away from the critical threshold. We elaborate on these three scenarios in detail in the next section.

## 3 THE ‘EXCESS RESOURCES ELONGATION’ AND ‘LAST RESORT ELONGATION’ HYPOTHESIS

Haussmann and Mauck (2008) proposed the ‘elongation hypothesis’ that explains TL elongation as an adaptive mechanism. It assumes that events of elongation of TL with age are positively associated with the ‘intrinsic phenotypic quality’ of individuals. The hypothesis assumes that certain individuals perform better than others (e.g., due to better early environment conditions or genetic advantages) and it is those individuals who are able to invest in telomere elongation. However, Haussmann and Mauck (2008) concluded that the pattern of TL elongation over time in their study appeared to be due to the selective disappearance of individuals with shorter TL and, hence, that there was no indication of a net increase in TL within individuals (although they lacked longitudinal data to directly test this idea). Given the increasing number of recent (including longitudinal) studies that suggest that telomere elongation occurs also later in life (see above, Table 1), we have recently argued that the elongation hypothesis needs to be reconsidered and we have renamed Haussmann and Mauck's original hypothesis to the ‘excess resources elongation’ hypothesis to contrast it with another hypothesis, the ‘last resort elongation’ hypothesis (Tobler et al., 2022). Here, we elaborate on these two hypotheses, relate the published studies reporting telomere elongation to the hypotheses and propose ways to test them.

The ‘excess resources elongation’ hypothesis (EREH; Tobler et al., 2022) extends the elongation hypothesis proposed by Haussmann and Mauck (2008). It assumes that only individuals with access to more/better resources (typically individuals that are high performers, i.e., of high intrinsic phenotypic quality sensu Haussmann & Mauck, 2008) can temporarily invest in telomere restoration mechanisms to such an extent that it results in elongation of TL because only those individuals are likely to cope with the associated costs of elongation (see above and Tobler et al., 2022). If telomere restoration mechanisms (and elongation in particular) demand resources (e.g., energy or nutrients) it needs to be traded off against other resource demanding functions (such as other maintenance functions or reproduction). Under these conditions, telomere elongation would primarily be expected in (i) individuals with high intrinsic quality (those with more access to limiting resources) or (ii) at times when there is an overall excess of resources when we predict that telomere elongation would occur in a rather large fraction of individuals in the population. Under the EREH, costs of telomere elongation are immediate, building on the idea of a typical short‐term life‐history trade‐off where energy or nutrients are limited. We can foresee two scenarios about the timing of telomere elongation in individuals with access to more resources under the EREH: (i) telomere elongation may occur *before* an anticipated period of life stress, as a ‘foresight’ of an anticipated period of accelerated telomere shortening (see e.g., Hoelzl et al., 2016), or (ii) telomere elongation may occur at some timepoint *after* a stressful period (that has inflicted considerable telomere shortening) when conditions are more benign (i.e., when resources are more plentiful and/or the workload is lower) allowing the individual to afford a larger investment in telomere restoration processes and, thus, to some extent ‘turn‐back‐the‐clock’ in terms of cellular ageing (see e.g., Asghar et al., 2018).

The ‘last resort elongation hypothesis’ (LREH; Tobler et al., 2022) also assumes that telomere restoration entails costs for individuals, but in this case, these costs are likely to be more substantial acting over longer terms and entailing more severe consequences, such as causing (severe) somatic damage and increasing the risk of contracting cancer. The idea behind the LREH is that due to the assumed potentially large costs associated with the activation of telomere restoration mechanisms, net elongation of TL is expected to occur only at a (life) stage when the risk of having too short telomeres outweighs the TL restoration costs. This would typically happen as a ‘last resort’ when telomeres have shortened to the critical point where further ‘normal’ shortening would take TL below the critical threshold and, thus, result in rapid cell senescence and organ dysfunction. In contrast to the EREH, the LREH predicts that a particular telomere length, rather than access to more resources, triggers telomere elongation. We might expect telomere elongation to occur primarily in older individuals and/or in (younger) individuals of relatively low intrinsic phenotypic quality, as these individuals are most likely to have so short telomere length that it is close to the critical threshold. In such individuals, telomere elongation may be expected as a type of ‘terminal investment’ increasing the chance that the individual will survive at least the ongoing life stage event (e.g., the current reproductive event). Typically, these types of individuals would be suffering severe TL shortening before upregulating their TL restoration processes to such an extent that it results in telomere elongation. Based on the assumptions of the LREH hypothesis, individuals with critically short telomeres would invest in surplus telomere restoration even if resource availability may be limited and/or their intrinsic phenotypic quality may be rather low, because direct costs (e.g., in terms of energy or nutrients) for this process are assumed to initially be relatively small. It is the induction of *severe delayed costs* (somatic senescence and effects of malign cancer) that are the key cost factors refraining individuals from regularly elongating their telomeres according to the LREH. But in a terminal investment situation such delayed costs are anyway unlikely to become ‘expressed’, because such individuals are likely to die beforehand. In older individuals whose TL slowly approaches a critically short length, telomere elongation may also be expected as an attempt to ‘escape’ the critical threshold. For older individuals that may be of high intrinsic quality this may be a way to (at least occasionally) live on even for several years/breeding attempts after having invested in repeated telomere elongation events to a point where telomere restoration costs have accumulated resulting in severe somatic damage or cancer.

**FIGURE 2:**
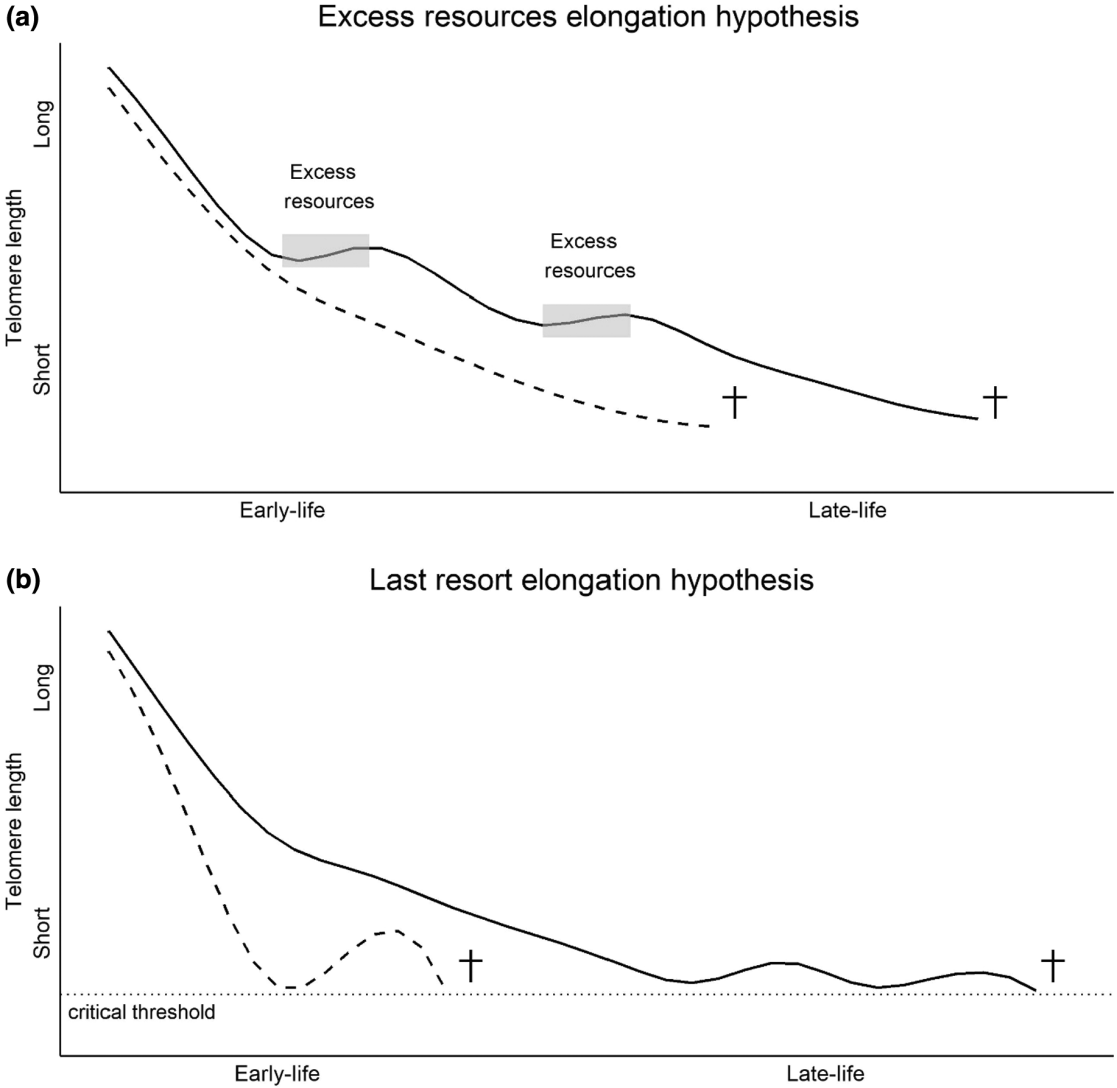
Hypothetical example of telomere dynamics with age and the predictions of when and which type of individuals that should be expected to activate their telomere restoration processes to such an extent that it results in net elongation of telomere length (TL). In (a), the ‘excess resources elongation hypothesis’ predicts that only high‐quality individuals (solid line) will have access to an excess of resource that allow them to afford to invest in costly telomere elongation. Meanwhile, poor‐quality individuals (dashed line) do not have access to these resources and therefore will not be able to invest in restoration to the extent that it results in any net elongation of TL. In (b), the ‘last resort elongation hypothesis’ predicts that telomere elongation is expected under two scenarios: (i) In individuals that are in a ‘desperate’ state (i.e., having such a short TL that it is close to the critical threshold after which further shortening trigger cell death). This ‘desperate’ state could be reached at an early chronological age by low‐quality individuals (dashed line) that have suffered high TL shortening rates and, thus, are expected to show large TL elongation. (ii) In later life by biologically older individuals (solid line), i.e., that have reached a life stage when they have generally short telomeres, that, even though they may be less exposed to TL degrading processes, still would benefit from limited elongations, e.g., prior to a new breeding period, to prevent them from reaching the critical threshold. This could be reiterated several times as costs for limited TL elongation may be small for these individuals that may be of higher quality and/or have access to high‐quality resources.

As they are outlined above, the EREH and the LREH hypothesis make different predictions regarding the timing when individuals should be expected to activate their telomere restoration processes and the type of individuals that should invest in elongation. Under the EREH it is predicted that (i) telomere elongation may occur at any time throughout life, whenever resource availability/physiological condition permits it, irrespective of the current TL of the individual, and (ii) that individuals of lower intrinsic phenotypic quality generally will not show telomere elongation (Figure 2a). In contrast, the LREH predicts that telomere elongation will occur when TL approaches the critical threshold towards the end of an individual's biological lifespan. Individuals that are in poor condition or of lower intrinsic phenotypic quality may reach this threshold faster, e.g., because they have a higher telomere shortening rate (Figure 2b). Thus, under the LREH individuals are facing a critical situation that directly affects the trade‐off between current versus future investments. The benefit of activating the telomere restoration pathway to elongate telomeres and, thus, to escape from the *immediate* severe somatic problems associated with a high risk of mortality, should exceed the costs, such as an increased risk of cancer or other soma‐damaging processes reducing later‐life performance. This may allow an individual to finish an ongoing, or even reach additional, reproductive events and, thus, have an overall positive effect on fitness.

Note that even though we contrast the two hypotheses above, they could be at work at different ages/life stages in the same individual. For example, individuals may experience benign conditions (e.g., access to excess resources) that may allow them to sometimes invest in telomere elongation during adult life, but they may also show telomere elongation when they later in life reach a TL that is close to the critical threshold. Another possibility is that the two hypotheses can combine if conditions become benign and favourable for a whole population (such as due to increases in ambient temperature and if food availability improves considerably in spring, after a winter period with harsh conditions). In such situations, we might predict that it is individuals with short telomere length (i.e., closer to the critical threshold) that will elongate their telomeres the most, whereas individuals with long telomeres may not or only slightly elongate their telomeres and instead invest more in other processes (e.g., replenish somatic stores or increase breeding effort including mate attraction behaviours).

## 4 CURRENT PATTERNS SUPPORTING THE EREH AND LREH

Over the last decade, the number of longitudinal studies reporting net increases in TL between time points in a non‐negligible proportion of individuals in a population has increased considerably (see Table 1). However, in several of these cases, the TL data have been analysed as a single quantitative process (i.e., telomere dynamics; e.g., Chen et al., 2011; Gardner et al., 2005; Kark et al., 2012). If analysed in this way, because the general pattern is that most individuals show telomere shortening (especially early in life or when comparing over a long period of their lives), cases of telomere elongation will merely be treated as ‘negative shortening’ with just small quantitative ‘subtraction’ effects on the magnitude of the shortening rate. Probably due to the lack of a scientific framework that deals specifically with telomere elongation in ecological and evolutionary studies, and because telomere elongation often has been viewed as a methodological artefact (see above), many previous studies have not explicitly discussed and even fewer specifically analysed telomere elongation patterns that appear to be present in their data set (e.g., Seeker et al., 2021; Sheldon et al., 2022; but see van Lieshout et al., 2019; Wolf et al., 2021; Voirin et al., 2023).

The EREH and the LREH both assume that telomere elongation entails non‐negligible costs. However, the type and magnitude of these costs differ between the two hypotheses (see above). As a consequence, we expect that the EREH and the LREH differ with respect to under what conditions telomere elongation will appear more frequently, and which type of individuals are expected to show elongation. Recently, several studies have shown telomere elongation in relation to factors such as food availability, infection status or proxies of individual condition/quality, in line with the patterns that would be expected by the EREH hypothesis (Brown et al., 2021; Gómez‐Blanco, 2023; Hoelzl et al., 2016; Wolf et al., 2021). In a study on free‐living edible dormice (*Glis glis*), animals artificially supplemented with sunflower seeds were capable of counteracting the degree of telomere shortening and elongate their TL during hibernation (a time of the annual cycle that appears to be benign in this species; Hoelzl et al., 2016). This suggests that only individuals with access to an excess of resources could afford to invest heavily in telomere restoration. Similarly, Brown et al. (2021) showed that in female Seychelles warblers (*Acrocephalus sechellensis*), individuals that had experienced more benign circumstances (e.g., lack of malaria infection, presence of helpers‐at‐the‐nest) showed telomere elongation and higher subsequent survival, in agreement with the EREH. In the latter case, however, it cannot be excluded that it was older individuals close to the critical threshold that elongated their telomeres and, thus, improved survival over several years (as predicted also by the LREH; see above). Furthermore, a recent study on barn swallows (*Hirundo rustica*), showing an increase in telomere length in chicks living in nests with experimentally reduced brood size where individual food‐intake can be assumed to be increased (Voirin et al., 2023), also supports the idea of telomere elongation occurring under more benign conditions. However, the same study found no relationship between telomere elongation and brood size in a subsequent year and another study on barn swallows also found no association between telomere elongation and indicators of nestling conditions (Parolini et al., 2015). Hence, more studies are needed that investigate the relationship between telomere elongation and resource availability/physiological condition.

In the case of the LREH, several studies on humans and wild birds appear to provide some support for the hypothesis (Bize et al., 2009; Elmore et al., 2008; Farzaneh‐Far et al., 2010; Gómez‐Blanco, 2023; Nordfjäll et al., 2009; Shalev et al., 2013; Svenson et al., 2011). In the latter studies that analysed telomere dynamics over time, negative correlations between the baseline TL measurement and telomere shortening were found, suggesting that individuals with the shortest telomeres are the ones that apparently show the highest incidence of telomere elongation (but note that it is hard to determine to what extent some of the early studies may be confounded by ‘regression to the mean’ effects; see e.g., Barnett et al., 2005). These results are consistent with the LREH prediction that telomere restoration mechanisms are activated particularly in individuals that may be in a ‘desperate’ state when their TLs are coming close to the critical threshold, and it would therefore be beneficial for them to elongate their telomeres.

## 5 CAN TELOMERE RESTORATION MECHANISMS BE ACTIVATED TO SUCH AN EXTENT THAT IT LEADS TO RAPID TELOMERE ELONGATION (IN ADULTS)?

Mechanisms for telomere restoration and elongation include the activity of the enzyme telomerase, recombination in the telomere regions, and transposable elements (Aubert & Lansdorp, 2008; Cenci, 2009; Dunham et al., 2000; Greider & Blackburn, 1985). Among vertebrates, the major mechanism of telomere restoration is the activation of telomerase (Blackburn et al., 2006; Shay, 2016). Telomerase is a polymerase that builds and attaches new telomeric repeats to the chromosomal ends, thus restoring TL and counteracting telomere shortening (Chow et al., 2012; Vega et al., 2003). The regulation of telomerase activity varies between different organisms, life stages and tissues (Hornsby, 2007; Wright et al., 1996). In many ectotherms and short‐lived homeotherms, telomerase has been shown to be active in different tissues throughout life, whereas in long‐lived homeotherms it is usually assumed that telomerase is active primarily in embryonic tissues and during early life (e.g., Frydrychová et al., 2023). Especially in adults of humans and other long‐lived vertebrates (e.g., non‐human primates, whales, bats), it has been shown that telomerase activity is mainly restricted to specific tissues that are undergoing rapid proliferation (e.g., germline, epithelial, and haemopoietic cells), whereas in most somatic cells there is typically no or only low levels of telomerase activation beyond the early stages of development (Bekaert et al., 2004; Foley et al., 2018; Gomes et al., 2011). In adults of other, typically short‐lived, non‐human vertebrates (e.g., small rodents, zebrafish), telomerase activation has been found also in somatic tissues suggesting that TL can be actively maintained or even elongated in these tissues (Anchelin et al., 2011; Gorbunova & Seluanov, 2009; Seluanov et al., 2007). However, these different telomerase activity patterns in short‐lived versus long‐lived species are an oversimplification. In birds, for example, where little is known about telomerase expression across life stages, Haussmann et al. (2007) compared telomerase activity in bone marrow across life stages in four bird species that differed markedly in life history and found that telomerase activity was as high in adulthood as at the nestling stage in long‐lived seabirds, and that also in a short‐lived songbird (the zebra finch) telomerase activity was still rather high at adulthood (albeit lower than at the nestling stage). Moreover, studies on humans also support the idea of transient activation of telomerase activity. Apparent lengthening of telomeres in somatic tissues has been found in several studies (e.g., Epel, 2012; Farzaneh‐Far et al., 2010; Huzen et al., 2014) and a recent study by Asghar et al. (2018) showed telomere elongation and increased telomerase activity in leucocytes within 1 year after a malaria infection.

Although telomerase activation is the major proximate mechanism for telomere elongation, it has been suggested that the apparent lengthening of telomeres may also come about through other processes such as increases in the proportion of ‘younger’ cells or redistribution of cell subpopulations, mechanisms that have been termed ‘pseudo‐lengthening’ (Epel, 2012). In these cases, changes in telomere length measurements are not due to actual lengthening of telomeres but because measurements are based on different cell subsets. In the first case, apparent elongation of telomeres in blood cells may be a consequence of increased cell turnover rate, for example, due to elevated levels of oxidative stress, that triggers a redistribution and replenishment of long‐telomere (i.e., young) cells from a haematopoietic repopulating pool (Epel, 2012; Shlush et al., 2011). This haematopoietic repopulating pool cell population is assumed to have more stable telomere length and to consist of less differentiated haematopoietic cells. Thus, if this mechanism is at work an increase in telomere length comes about through the activation of different subpopulations of haematopoietic stem cells in the bone marrow rather than the addition of telomeric repeats to the ends of chromosomes. In the second case, changes in telomere length may also come about through redistribution of cell subpopulations. For example, changes in the percentages of circulating immune cell subsets (e.g., as a result of an infection) may result in an average increase in leucocyte telomere length. Pseudo‐lengthening through cell subset redistribution is more likely to occur in measurements based on mammal blood where only leucocytes are nucleated. However, even in taxa that have nucleated blood cells such as birds or reptiles, changes in the percentages of red blood cells versus immune/white blood cell types may cause a shift in telomere length measurements if based on whole blood. It has been shown that immune cell types in Australian painted dragon lizards (lymphocytes and azurophils) have 270% and 388% longer telomeres than red blood cells (Olsson et al., 2020), and changes in their relative proportions could potentially affect average blood cell telomere length. However, telomere elongation observed when measurements are solely based on nucleated red blood cells (e.g., Xiong, 2023, see also Table 1) is unlikely to be due to redistribution of immune cell subsets. However, critical to the ‘telomere elongation’ hypotheses discussed in the present article, ‘pseudo‐elongation’ of telomeres is also a biological phenomenon and therefore likely to be associated with increased short‐ or long‐term costs, and thus affect Darwinian fitness. If telomere elongation is due to a major shift in the composition of immune cells it would reflect, e.g., infection status and, thus, would be associated with the costs of activating the immune system and exposure to increased levels of oxidative stress (e.g., von Schantz et al., 1999). If the apparent elongation is a result of replenishment of young cells, this may lead to more rapid depletion of stem cell sources (Epel, 2012) and consequently result in an onset of accelerated cell and tissue ageing and thus may lead to earlier onset of senescence.

## 6 HOW CAN THE EREH AND LREH BE TESTED?

One obvious approach to test the EREH is to experimentally manipulate resource availability (e.g., food supply or nutrition quality) for a group of individuals. If telomere elongation is resource dependent, according to the EREH it is predicted that individuals given excess resources should show a higher occurrence of telomere elongation. Concomitant experimental manipulation of resource availability and telomerase expression (e.g., through the use of TA‐65, a plant extract from *Astragalus membranaceus* and presumed activator of telomerase; Criscuolo et al., 2018; de Jesus et al., 2011; Reichert et al., 2014) in a 2 × 2 experimental design would make it possible to evaluate whether individuals of high intrinsic phenotypic quality/high resource access would elongate their TL independently of the manipulation of telomerase activity. Under the EREH, one would predict that the group receiving both extra resources and a stimulant of telomerase activity would have the highest proportion of individuals with elongation, whereas the group with limited resource availability would show the lowest proportion of individuals with elongation. It would also be plausible that a ‘forced’ activation of telomerase with TA‐65 may incur increased costs to individuals with limited access to resources that they cannot afford, which may result in increased mortality. Such a finding would support the EREH which predicts that telomere elongation under circumstances that are not favourable leads to subsequent costs in terms of lower survival. Detecting cancer in wild animals is a challenging task, however, among those individuals forced to activate the telomere elongation pathways, we can study the activity of genes related to cancer. By monitoring gene expression, we can explore possible connections between telomere elongation and the risk of cancer development, and its long‐term cost (Giraudeau et al., 2019; Ujvari et al., 2022). Indirect indications in favour of the EREH hypothesis can also be based on correlational studies. The EREH predicts that only high intrinsic phenotypic quality individuals that have excess resources show elongation and it can therefore be investigated if measures of body condition (or other species‐specific phenotypic quality indicator traits) show positive relationships with the occurrence of telomere elongation.

Experimental breeding designs could also provide data to test the LREH hypothesis. One could, for example, select individuals with (very) short and (very) long telomeres and expose half of the individuals from each of these two groups to experimental treatments that induce stress or increase workload (for example increasing brood size in breeding birds or inducing physiological stress through injection of immune agents or stress hormones). According to the LREH hypothesis, one would expect that individuals with short telomere lengths would be more likely to show telomere elongation than individuals with long telomeres when exposed to extra workload or stress. Moreover, if telomere shortening is a physiological cost of reproduction (see Sudyka, 2019 for a detailed discussion) that can lead to rapid telomere shortening and push organisms closer to the critical threshold in TL, it might be expected that telomere elongation occurs more frequently in non‐breeders or after breeding is complete. According to the LREH hypothesis, (heavy) reproductive investment might ‘force’ individuals coming close to the critical threshold to elongate TL to compensate for the incurred telomere loss, at the expense of reducing/skipping future reproductive investments. This reasoning is congruent with the recently proposed idea that telomerase may be a pleiotropic regulator of the life‐history trade‐offs between growth, somatic maintenance and reproduction (Frydrychová et al., 2023).

The LREH can also be investigated using correlational approaches. Under the LREH, the individuals that are more likely to exhibit elongation are those at the short end of the telomere distribution in the population. So, it could be tested if within a given population there is a pattern of more frequent telomere elongation (and telomerase expression) in individuals with relatively short TL (e.g., individuals that belong to the shortest TL quartile year_*n*−1_). Likewise, this expectation could be approached with longitudinal studies comparing the relative importance of telomere shortening rate versus coming close to the critical threshold in TL, and to do such comparisons between individuals with different life spans within a species. However, in such studies where a baseline telomere length (at time *t*) is related to the change in telomere length (increase or decrease in TL between *t* and *t* + 1), ‘regression to the mean’ effects need to be accounted for (e.g., Barnett et al., 2005; Kelly & Price, 2005). For example, this can be done by measuring telomere length at three time points (where the first two could be relatively close in time) and relate the change in telomere length between the second and third measurement to the telomere length measured at the first time point (Barnett et al., 2005). However, note that it may be difficult to test the LREH using the correction method suggested in Verhulst et al. 2013 because this correction of change in telomere length is based on the correlation coefficient between the first and the second measurement and it assumes that it is only reflecting measurement error (and thus not reflecting any biologically relevant change in telomere length) that defines the value of the correlation coefficient. Hence, if the change in telomere length between the two time points is caused by any biologically relevant factor (and not only measurement error), this correction method would consider all deviation from 1 in the correlation coefficient as measurement error and thus ‘correct away’ also all biologically relevant changes that have happened between the two time points. This would have a particularly strong impact on the correction of the telomere length change for the shortest and longest measures of telomere length at the first measurement occasion.

In addition to linear analyses using the net change in telomere length between time points as a continuous variable, it could also be interesting to conduct complementary analyses splitting the dataset into ‘shorteners’ and ‘elongators’. Although dichotomization of continuous variables should be treated with caution, such binary analyses could provide further insight into how telomere elongation per se is associated with particular life‐history strategies or behavioural phenotypes (e.g., slow or fast pace‐of‐life individuals).

Evaluating telomere elongation hypotheses in free‐living individuals is challenging because extensive longitudinal data are required to detect subtle changes in TL. In order to generate data that could provide stronger tests of the EREH and the LREH, it is critical to collect longitudinal data where telomeres are measured several times over the lifespan of an individual, especially before and after key life‐history stages (including at late life stages), and to conduct such studies both in short‐lived and long‐lived species. To address these challenges, advanced methods for measuring telomere length, such as single telomere length analysis (Baird et al., 2003), can provide greater precision at the level of individual telomeres. This approach may also provide further insights into the mechanistic basis of telomere elongation (e.g., whether telomere elongation is due to telomerase activity or ‘pseudo‐elongation’). Together with experimental manipulation of TL as described above, it could be a promising approach to further explore telomere elongation and help establish causal links between telomere elongation and its short‐ and long‐term costs and benefits.

## 7 CONCLUSION

We argue that patterns of (net) elongation of telomere length deserve more attention, including targeted investigations from ecologists and evolutionary biologists, and we propose that patterns of telomere elongation may be rather different in origin than patterns of telomere shortening. Episodes of transient telomere elongation may be of particular interest as they could represent individual decisions to optimise life‐history strategies and maximise fitness, which may not be reflected in the overall telomere dynamics pattern. Particularly interesting questions are whether telomere elongation is restricted to certain periods of life (e.g., less demanding periods, such as the non‐breeding season or periods with benign climate/weather), if it involves certain types of individuals (e.g., those of high intrinsic phenotypic quality, without infections or older individuals), or if it occurs when a certain length of the telomere is reached (e.g., close to the critical threshold when TL becomes so short that cell senescence may set in). Moreover, phenotypic variation in telomere elongation across species, populations and among individuals within populations, could also be linked to pace‐of‐life syndromes (Marasco et al., 2022; Ricklefs & Wikelski, 2002; Réale et al., 2007). According to the pace‐of‐life syndrome hypothesis, behavioural phenotypes can be grouped into ‘fast’ pace‐of‐life individuals that adopt a ‘live fast‐die young’ strategy (with little or no investment in somatic maintenance) and ‘slow’ pace‐of‐life individuals (with higher levels of somatic maintenance, Réale et al., 2010; see also Criscuolo et al., 2021, Giraudeau et al., 2019). Based on this hypothesis, we expect less restoration, and thus low/no incidence of elongation, in individuals with a fast pace‐of‐life strategy, and more frequent restoration, and thus a higher incidence of elongation, in individuals adopting a slow pace‐of‐life strategy. Specific analyses of telomere elongation will reveal whether these events reflect individual strategies to optimise life histories.

Even though the mechanistic underpinnings of telomere elongation are not well understood, they may be similar to those acting in ordinary telomere restoration processes (where telomere shortening can be ‘slowed down’ by telomere restoration mechanisms). Organisms may show telomere (net) elongation at certain points in life which can be seen as an ‘over‐compensation’ of telomere restoration. This ‘over‐compensation’ is surprising given that there are strong reasons to believe that there are some non‐negligible and even possibly substantial costs involved in (prolonged) activation of the telomere restoration processes. This makes it very interesting to in detail study why and when organisms are willing to take the risk to not only compensate for TL loss but even activate their TL restoration mechanisms to such an extent that it results in a (temporary) net increase in TL. In future studies, investigating telomere elongation as a genuine phenomenon could offer insights into its potential role as an adaptive behaviour. We hope that the hypotheses framework described in this article will encourage more research that focuses on telomere elongation and the potential costs associated with it.

## AUTHOR CONTRIBUTIONS

**David Gómez‐Blanco:** Conceptualization (equal); validation (supporting); writing – original draft (lead); writing – review and editing (supporting). **Michael Tobler:** Conceptualization (equal); supervision (supporting); validation (equal); writing – review and editing (equal). **Dennis Hasselquist:** Conceptualization (equal); funding acquisition (lead); supervision (lead); validation (equal); writing – review and editing (equal).

## CONFLICT OF INTEREST STATEMENT

We declare we have no competing interests.

## Data Availability

This document does not involve the utilization of any data.
